# Individually Adapted Imagery Improves Brain-Computer Interface Performance in End-Users with Disability

**DOI:** 10.1371/journal.pone.0123727

**Published:** 2015-05-18

**Authors:** Reinhold Scherer, Josef Faller, Elisabeth V. C. Friedrich, Eloy Opisso, Ursula Costa, Andrea Kübler, Gernot R. Müller-Putz

**Affiliations:** 1 Institute for Knowledge Discovery, Graz University of Technology, 8010 Graz, Austria; 2 BioTechMed-Graz, Austria; 3 Clinic Judendorf-Straßengel, 8111 Gratwein-Straßengel, Austria; 4 Department of Cognitive Science, University of California, San Diego, La Jolla, CA, 92093, USA; 5 Institut Guttmann, Institut Universitari de Neurorehabilitació adscrit a la UAB, 08916 Badalona, Barcelona, Spain; 6 Institute of Psychology, University of Würzburg, 97070 Würzburg, Germany; UNIVERSITY OF ROME TOR VERGATA, ITALY

## Abstract

Brain-computer interfaces (BCIs) translate oscillatory electroencephalogram (EEG) patterns into action. Different mental activities modulate spontaneous EEG rhythms in various ways. Non-stationarity and inherent variability of EEG signals, however, make reliable recognition of modulated EEG patterns challenging. Able-bodied individuals who use a BCI for the first time achieve - on average - binary classification performance of about 75%. Performance in users with central nervous system (CNS) tissue damage is typically lower. User training generally enhances reliability of EEG pattern generation and thus also robustness of pattern recognition. In this study, we investigated the impact of mental tasks on binary classification performance in BCI users with central nervous system (CNS) tissue damage such as persons with stroke or spinal cord injury (SCI). Motor imagery (MI), that is the kinesthetic imagination of movement (e.g. squeezing a rubber ball with the right hand), is the "gold standard" and mainly used to modulate EEG patterns. Based on our recent results in able-bodied users, we hypothesized that pair-wise combination of "brain-teaser" (e.g. mental subtraction and mental word association) and "dynamic imagery" (e.g. hand and feet MI) tasks significantly increases classification performance of induced EEG patterns in the selected end-user group. Within-day (How stable is the classification within a day?) and between-day (How well does a model trained on day one perform on unseen data of day two?) analysis of variability of mental task pair classification in nine individuals confirmed the hypothesis. We found that the use of the classical MI task pair hand vs. feed leads to significantly lower classification accuracy - in average up to 15% less - in most users with stroke or SCI. User-specific selection of task pairs was again essential to enhance performance. We expect that the gained evidence will significantly contribute to make imagery-based BCI technology become accessible to a larger population of users including individuals with special needs due to CNS damage.

## Introduction

Some mental activities induce changes in spontaneous electroencephalogram (EEG) rhythms in a very specific and predictive way. This means that an individual can generate distinct EEG patterns at will and independently from sensory stimulation. Brain-computer interfaces (BCIs) detect such EEG patterns and translate them into action. See [1–6] for a review on BCI technology.

The majority of modern imagery-based BCIs utilize motor imagery (MI) to encode messages (e.g. [4, 7–16]). MI, that is the kinesthetic imagination of movement, induces transient changes in sensorimotor EEG rhythms. More precisely, MI results in amplitude suppression (event-related desynchronization, ERD) or enhancement (event-related synchronization, ERS) in specific oscillatory components over defined brain areas [17]. The literature rarely provides very specific details on the MI tasks individual users perform. Common MI tasks are the kinesthetic imagination of movements of the left or right hand (e.g. wrist extension and flexion or squeezing movements) or both feet (e.g. dorsiflexion or foot pedal pressing tasks). We typically ask users whether they have preferred movements or whether they are familiar with specific movements from daily activities (e.g. sport-related activities or playing a musical instrument). Once movements are identified, subjects are usually asked to repetitively perform the mental motor task at a comfortable speed for a given period of time with the aim to induce sustained ERD and/or ERS patterns. Note that users are asked to keep their attention on the MI task and avoid imagining very fast or very slow movements. The issue is to prevent users from imagining automated motion sequences or successions of individual isolated movements. In both cases, (sub)cortical neural networks are activated in different ways, which may result in discontinuous ERD and/or ERS patterns (for example, mu rhythm ERD is followed by beta ERS (rebound) after end of individual movement). This is in line with the finding that sensory motor rhythm BCI performance correlates with prefrontal activation [18].

Operating mental imagery-based BCIs is a skill that has to be trained [3, 19, 20]. Users need to learn to generate EEG patterns reliably (feedback or reinforcement learning) for the machine to be able to translate them correctly (machine learning). Conventional training methods, however, often do not lead to the desired success (“BCI inefficiency”) [12, 21–23]. Discrimination between two distinct MI tasks is < 70% in about 40% of users [12]. There is common agreement that accuracy below 70% does not allow useful BCI operation [24]. Non-stationarity and inherent variability of EEG is one major issue for pattern classification: EEG signals typically change over time and EEG patterns are user-specific. Data-based time-invariant models are commonly used to characterize time-variant EEG [4, 25–27]. Various methods including time-invariant subspace decomposition, online co-adaptation and transfer learning are currently being examined to enhance classification performance [15, 28–31]. First results of these novel approaches are encouraging. Parallel to studying machine learning aspects of BCI to enhance performance, we have been investigating EEG pattern generation. We showed that kinesthetic imagery induces patterns that are more distinct and result in higher classification performances, when compared to the use of visual imagery of movements [32]. Furthermore, we found that the use of hand vs. feet MI leads to higher classification performances compared to the use of left hand vs. right hand MI tasks [33, 34]. Encouraged by the result that mental task choice impacts on pattern recognition performance, we recently started exploring possible alternatives to MI. Besides the use of MI, the literature reports on the use of e.g. mental mathematics, spatial navigation or object manipulation for operating a BCI (e.g. [7, 35–37]). Since the already mentioned mental tasks activate spatially distinct cortical areas, we hypothesized that an appropriate pair-wise combination of mental tasks inherently leads to highly discriminable EEG patterns [38]. Firstly, in off-line simulations [38–40] and recently, in online studies [37, 41], we were able to confirm our hypothesis. Key to enhance performance was the combination of “brain-teaser”, i.e., tasks that require problem specific mental work (e.g. mental subtraction or word association), and “dynamic imagery” tasks (e.g. MI or spatial navigation) [38], as well as subject-specific selection of task combination [37].

The mental task pair studies presented above were conducted in able bodied users in lab environments. Our primary goal in the context of BCI, however, is to develop novel augmentative communication applications and motor function restoration for individuals with functional disability (e.g. [11, 31, 42–45]). Functional disability means any long-term limitation in activity resulting from central nervous system (CNS) tissue damage. Highest priority is to provide them with a switch function, i.e., a reliable binary control signal. The goal of this study is to provide baseline evidence that appropriate pair-wise combination of mental tasks leads to enhanced EEG pattern discrimination in users with functional disability. The performance in this end-user group is typically lower than the performances able-bodied users achieve (< 70%, e.g. [46]).

## Results

**Fig 1:**
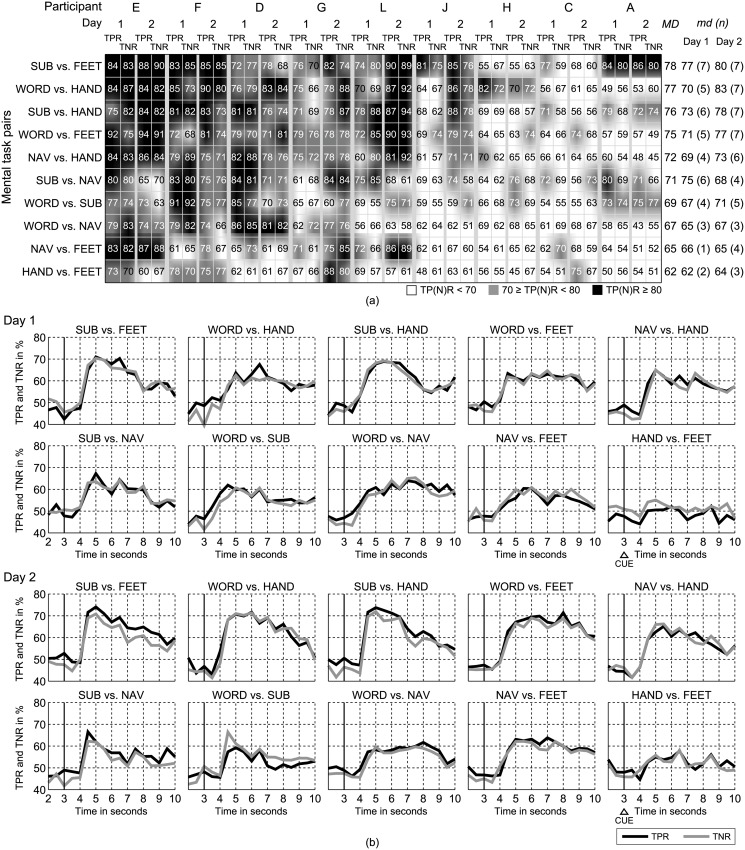
Within-day variability results. (a) Cross-validated within-day TPRs and TNRs. Peak TPR and TNR values, respectively, for the segment *S*_*t*_ = [*t*−1 *t*] with the highest geometric mean accuracy $GMAC=\sqrt{TPR\cdot TNR}$ during the imagery time period (*t* = [3.0, 3.5, …, 9.5]*s* after trial on-set) are presented. For better interpretation of the results, the rows and columns are sorted according to the median *GMAC* over class-pairs and day (1 and 2), and median over participants (user ID: A-L), respectively. Table entries are subdivided into three different levels of performances and color-coded. The last three columns list median *GMAC* values for each class-pair. The first column shows the median *GMAC* over subjects and days (*MD*). Column two and three list the median *GMAC* (*md*) and the number of subjects (n) with *GMAC* > 70% for each day. (b) Mean *TPR* and *TNR* curves, averaged over all subjects, for each day and mental task pair individually. The visual cue providing the information on the mental task to be performed was presented at *t* = 3*s* (vertical line).

To establish a baseline, we recorded multi-channel EEG while participants performed five distinct mental tasks on two different days. Mental tasks included word association (WORD), mental subtraction (SUB), spatial navigation (NAV), MI of the right hand (HAND), and MI of both feet (FEET). User details and the number of trials with artifacts that were excluded from the analysis are summarized in Table 1. Within-day and between-day variability of pair-wise single-trial mental task classification was investigated by offline simulation (10-times 10-fold cross validation). Within-day variability was assessed by ranking the discriminability of mental task pairs for each day separately. Peak true positive rate (TPR) and true negative rate (TNR) values in the range of 43–94% were calculated for individual subjects (Fig 1(a)). The temporal dynamics of TPR and TNR detections are shown in Fig 1(b). To favor a balanced classification performance, imagery pairs are ranked based on the geometric mean accuracy $GMAC=\sqrt{TPR\cdot TNR}$. With a median GMAC over participants *md* ≥ 77% on both days the class combination SUB vs. FEET achieved the highest single-trial classification performance. For seven out of the nine participants' peak *GMAC* > 70% (range 70–83%) were found on both days. Performances for mental task pairs WORD vs. HAND, SUB vs. HAND, and WORD vs. FEET were *md* ≥ 70% and *md* ≥ 77% on day one and two, respectively. The mental task combination HAND vs. FEET performed worst in average on both days (*md* ≤ 64%). For this pair *md* > 70% was calculated only for 4 out of 9 subjects (day 1: subject E, 71%; F: 74%; day 2: F, 76%; G, 84%; C, 71%;). There was a statistical significant difference in performance depending on the mental task pairs involved (Friedman Test, *X* ^2^(9) = 25.95, *p* = 0.0021). Post-hoc analysis with Wilcoxon signed rank tests was conducted with a Bonferroni correction applied, resulting in a significance level set at *p* = 0.05/45 = 0.0011 (pair-wise comparison of performance of 10 mental task pairs results in 45 combinations). Median GMAC over participants and days (*MD*) are listed in Fig 1(a). We found that both SUB vs. FEET (*Z* = −3.375, *p* = 0.0007) and WORD vs. HAND (*Z* = −3.288, *p* = 0.0010) performed significantly better than HAND vs. FEET, as well as that WORD vs. FEET performed better than NAV vs. FEET (*Z* = −3.332, *p* = 0.0009).

**Table 1:** Participant details. The ID, gender (Gndr), age in years, months (Mth) since occurrence and the type of event are shown for each individual. The number of trials with artifacts excluded from the analysis are listed for day 1/day 2 for each mental task. The last column lists EEG channels with artifacts that were excluded from analysis.

**Participant details**					**Artifacts**						
ID	Gndr	Age	Mth	Event	WORD	SUB	NAV	HAND	FEET	Total	Channels excluded
A	M	42	6	Locked-in syndrome due to brainstem stroke	6/8	12/4	5/6	7/3	6/5	37/28	AFz, F7, F6, T3, P7
C	F	31	5	Locked-in syndrome due to brainstem stroke	4/9	3/4	3/10	1/7	2/11	14/43	AFz, F7, F6, T4, PO3, O1
D	F	33	2	Spinal cord injury C5, ASIA C	1/2	6/3	3/4	1/3	1/3	13/19	AFz, F7, F6, PO4
E	F	40	255	Spinal cord injury C5, ASIA A	8/1	6/6	7/9	19/8	16/12	57/38	AFz, F7, F6, T3, T4, P7, P8, PO4
F	F	57	5	Massive hemorrhagic stroke in left hemisphere	3/2	0/3	2/5	0/3	1/4	7/19	F7, F6, T3
G	F	43	27	Spinal cord injury C5, ASIA C	2/6	5/3	5/4	5/5	3/5	21/25	F7, F6, C4, P6
H	F	20	6	Hemorrhagic stroke parietotemporal, right central no cranium	2/3	8/1	6/2	4/2	2/0	23/10	AFz, F7, F6, T3, P7
J	M	36	53	Spinal cord injury C5, ASIA A	7/4	4/5	6/8	11/9	9/6	38/34	AFz, F7, F4, F6, FC3, FC4, T4, P7, P8
L	M	38	15	Spinal cord injury C4, ASIA A	4/9	5/4	4/6	4/7	7/4	25/32	AFz, F7, T3, T4

**Fig 2:**
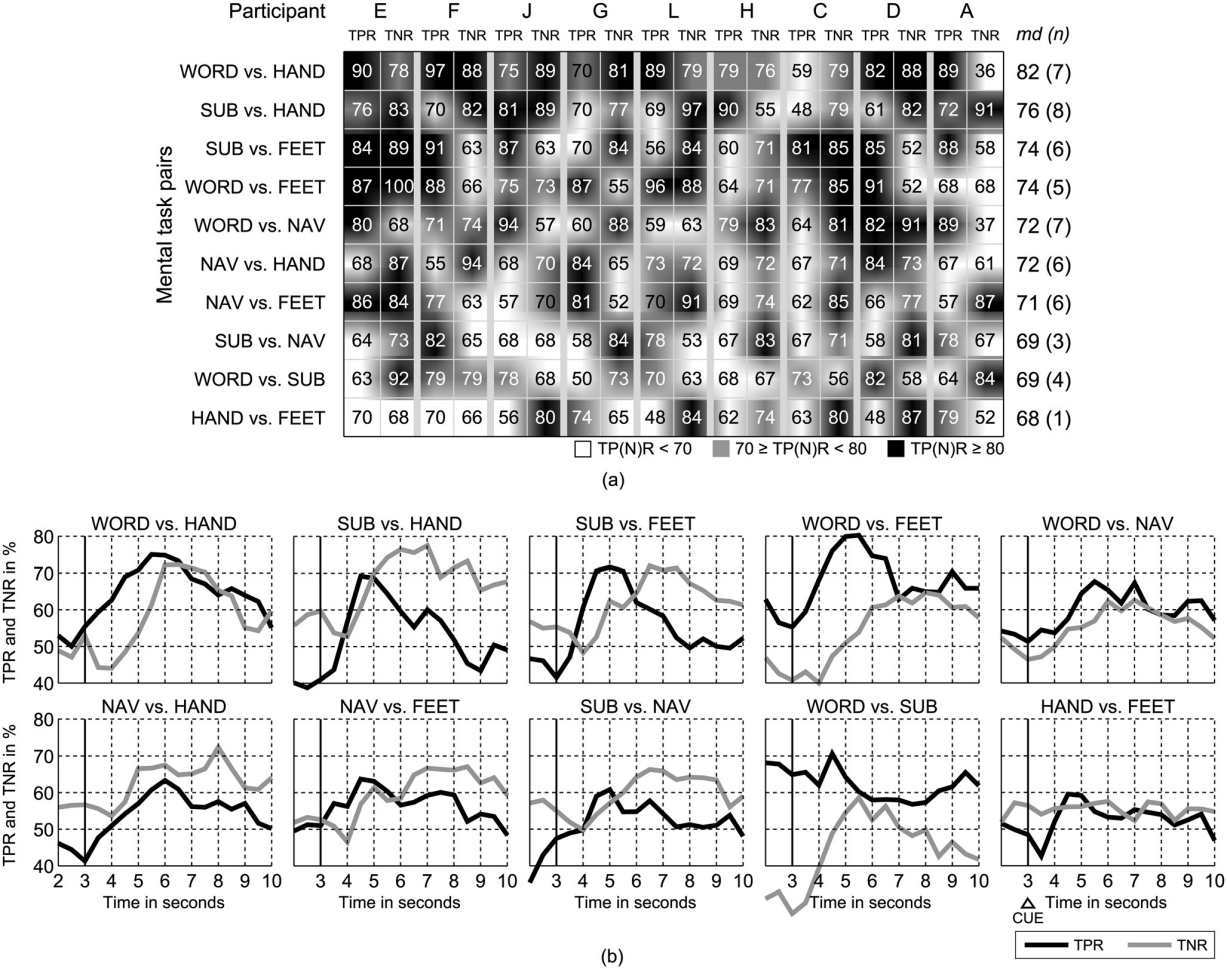
Between-day variability results. (a) Simulated peak *TPR* and *TNR* values (color coded), respectively, for the segment *S*_*t*_ with the highest $GMAC=\sqrt{TPR\cdot TNR}$ during the imagery period (*t* = [3.0, 3.5, …, 9.0]*s*). The column on the right list the median *GMAC* (*md*) and the number of subjects (n) with *GMAC* > 70%. (b) Mean TPR and TNR curves for day two, averaged over all participants, for each mental task pair individually.

Between-day variability was assessed by training BCI models for each participant with data from day one and by applying the model on day two. To mitigate the problem of EEG non-stationarity between days, the classifier bias was adapted from the first few trials of day two [47]. Fig 2(a) summarizes peak TPR and TNR. Corresponding curves are summarized in Fig 2(b). With *md* = 82% and seven out of nine subjects performing better than *GMAC* > 70% the mental task combination WORD vs. HAND achieved the highest overall performance. Worst performance was calculated for HAND vs. FEET (*md* = 68%). Only for one participant the performance threshold of *md* > 70% was exceeded. Achieved performances vary among users.

## Discussion

Both within-day analysis and between-day simulation confirm our hypothesis: Individual selection of mental task pairs significantly boosts binary classification accuracy of induced EEG patterns in end-users with functional disability.

### Motor imagery tasks

The literature shows that the use of HAND vs. FEET mental tasks pairs leads to binary accuracy < 70% in about 40% of able-bodied first-time BCI users [12]. Initial classification performance in users with functional disability is typically lower and improves with training (e.g. [13, 42]). The results of this study support such an initial performance distribution. There can be several reasons why the use of MI leads to performance slump in users with functional disability. One explanation could be that central nervous system (CNS) tissue damage results in changes in structural connectivity that lead to unspecific activity and hence to very similar EEG patterns. A different explanation could be the enjoyment of performing MI. After each experiment, participants were asked about the ease and enjoyment of performing the different mental tasks. The analyses of self-reports showed that participants enjoyed motor tasks less than non-motor tasks [48]. The use of MI of affected body parts may cause some adverse effects on imagery (for example, frustration due of the inability to move). A recent study in able-bodied individuals suggests that the sensation of body ownership makes MI become “easier” [49]. Moreover, sensation of body ownership has a positive impact on BCI performance [50]. This, however, also raises the question on whether or not body ownership is fundamental for good MI-BCI performance? Individuals with motor disability may neglect or have lost the sensation of body ownership/agency over affected muscles. As a consequence, motor imagery of affected body parts may not be best strategy for gaining reliable BCI control without extensive training. Addressing this question is important future work and will help gaining a better understanding of the underlying neural processes. The result that MI tasks pairs induce EEG patterns that are suboptimal in terms of pattern classification does not mean that MI should no longer be used. Results of this study and from the literature show that MI leads to accuracy > 70% in some BCI users with functional disability. Moreover, for applications such as BCI-based stroke motor function rehabilitation the use of MI is most appropriate and reasonable [51–53].

### “Brain-teasers” vs. “dynamic imagery” mental tasks

The increase in binary classification performance that results from using a combination of “brain-teaser” and “dynamic imagery” tasks as well as an individual selection of tasks is in line with previous offline and online studies with able-bodied users [37, 38, 40]. In general no clear differences in the computed performances between individuals with SCI and stroke were observed. We conclude that selecting user-specific mental task pairs enables a large number of individuals to benefit from BCI technology.

### Between-day model transfer

The between-day variability analysis clearly shows different dynamics of the distinct mental tasks. Specifically for the best performing tasks WORD and SUB, which peak about 2 seconds earlier when compared to HAND and FEET, respectively (Fig 2(b)). This is a result of the selected model transfer procedure. We trained our BCI model with features extracted from 1-s EEG segments on day one. Incorporating more information on temporal dynamics will further enhance the prediction performance and reduce misclassification. However, at the cost of timely feedback presentation. We typically try to minimize feedback delays to support reinforcement learning in users. One open question in the context of temporal dynamics is whether “more frequent but less accurate” (update rate > 10 *Hz*) or “less frequent but more accurate” (≤ 1 *Hz*) feedback is more beneficial for BCI skill acquisition.

Note that for the between-day variability analysis we were interested in examining the maximum performance that can be achieved when transferring the BCI model between sessions. Several classifiers were computed for each mental task pair. The results reported summarize the maximum performance achieved by an individual classifier on unseen data of day two. The common approach for BCI is to select parameters on day one that achieve maximum classification performance and apply them to day two. Evaluation of the simulation results when applying the maximum performance criterion for parameter selection, however, led to accuracies ≥ 80% only in 4 out of the nine participants on day two. We emphasize this drop in performance issue because our between-day model transfer simulation results suggests that the common maximum classification performance strategy is not optimal. Finding more appropriate selection criteria for parameter optimization is of utmost importance and needs closer addressing in the future.

## Conclusion

To conclude, in this study we systematically examined the impact of mental task choice on the performance of mental imagery-based BCIs in individuals with CNS tissue damage. The results of the study support that the choice of mental task significantly impacts on the classification performance in first-time imagery-based BCI users with functional disability. And, although motor imagery is the “gold standard”, the classification between hand vs. feet performs well only in a minority of users without training. The use of “brain-teaser” and “dynamic imagery” mental task combinations leads to significant performance increase in the majority of end-users with functional disability. Furthermore, the performance level that end-users with functional disability achieve is comparable to the performance that able-bodied users achieve.

## Methods

### Participants

Nine individuals with severe motor disabilities (seven female, age range 20–57 with a median age of 38, SD = 10) consented to participate in this study. The study, including the measurement protocol and the consent procedure were approved by the local ethics board, “Comitè d’Ètica Assistencial de l’Institut Guttman”. All participants gave informed, oral consent. In addition, written consent was obtained for every participant. The signed consent forms are stored with the participants’ clinical files. In many cases, written consent had to be provided by the participants’ legal representatives as many participants were not able to write due to tetraplegia. Details of the participants are summarized in Table 1. Participants attended a rehabilitation program at the Guttmann Institute in Barcelona, Spain, were naïve to the task and did not receive BCI training before participating in this study.

### Recordings

EEG was recorded from 30 electrode channels placed on the scalp according to the international 10–20 system. Electrode positions included channels AFz, F7, F3, Fz, F4, F8, FC3, FCz, FC4, T3, C3, Cz, C4, T4, CP3, CPz, CP4, P7, P5, P3, P1, Pz, P2, P4, P6, P8, PO3, PO4, O1, and O2. Reference and ground were placed at the left and right mastoid, respectively. Additionally, electrooculographic (EOG) activity was recorded from two electrodes placed on the outer canthus of the left eye and above the nasion. The g.tec GAMMAsys system with g.LADYbird active electrodes and two g.USBamp biosignal amplifiers (Guger Technolgies, Graz, Austria) was used for recording the biosignals. Biosignals were band pass filtered 0.5–100 Hz (notch filter at 50 Hz) and sampled at a rate of 256 Hz.

### Experimental paradigm

**Fig 3:**
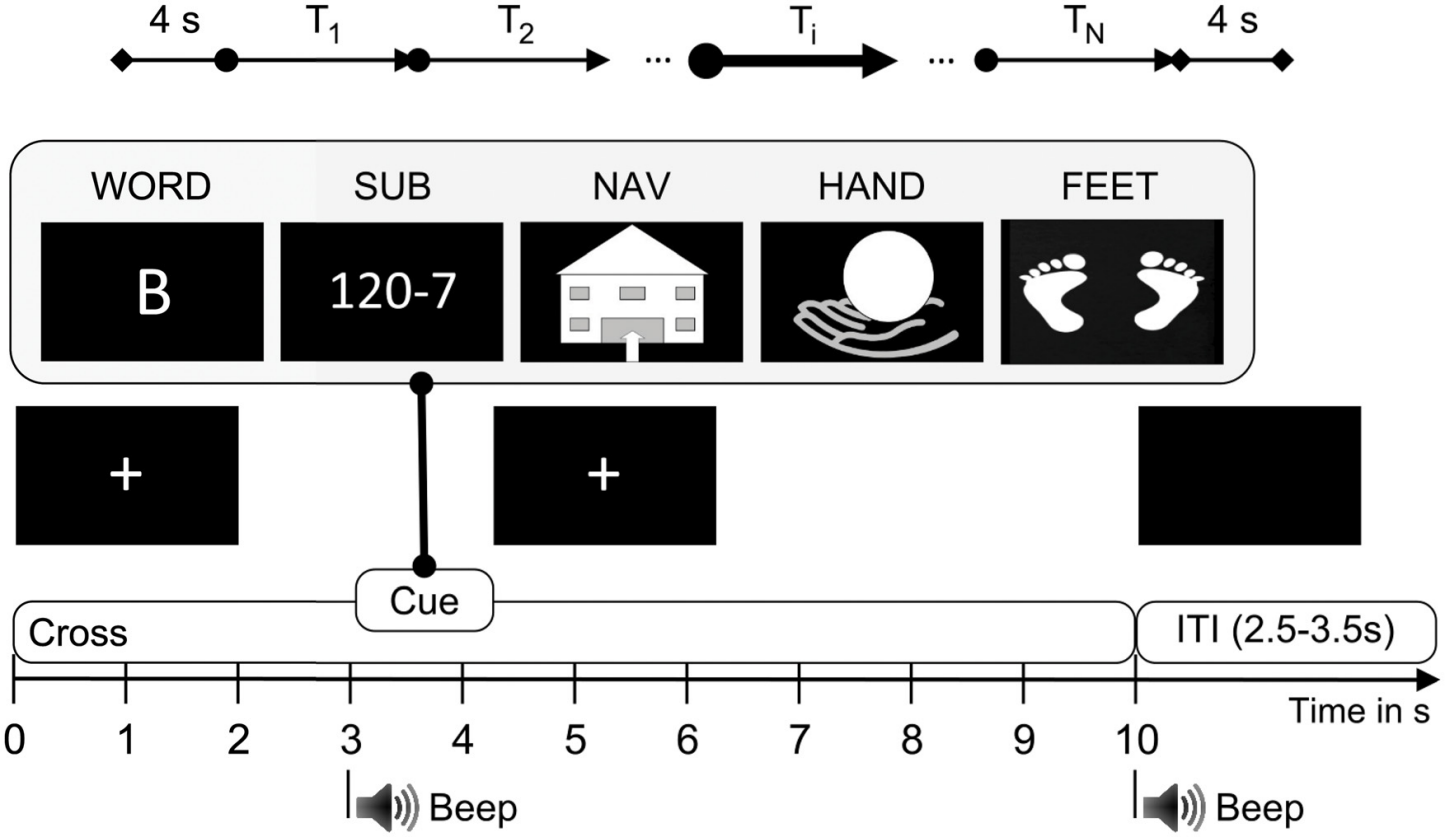
Experimental paradigm. The duration of single imagery trials (*T*_*i*_) was 10 s. At t = 0 s, a cross was presented in the middle of the screen. Participants were asked to relax and fixate the cross to avoid eye movements. At t = 3 s, a beep was sounded to get the participant’s attention. The cue indicating the requested imagery task, one out of five graphical symbols, was presented from t = 3 s to t = 4.25 s. At t = 10 s, a second beep was sounded and the fixation-cross disappeared, which indicated the end of the trial. A variable break (inter-trial-interval, ITI) lasting between 2.5 s and 3.5 s occurred before the start of the next trial. Participants were asked to avoid movements during the imagery period, and to move and blink during the ITI. Experimental runs began and ended with a blank screen (duration 4 s). Modified from [38].

The experiment was conducted on two different days with at least 5 days in between within a two-week period. Details on the cue-guided experimental paradigm are summarized in Fig 3. The screening session for a single subject consisted of 8 runs resulting in 40 trials of each class for each day. One single experimental run consisted of 25 cues, with 5 of each mental task. Cues were presented in random order. Participants were asked to continuously perform the specified mental imagery task for 7 seconds. Mental tasks included:

- Word association (WORD): Generation of as many words possible beginning with the presented letter in Spanish language (e.g. B = bank, bold, buy, etc). Letters were presented in pseudo-randomized order.
- Mental subtraction (SUB): Calculation of successive elementary subtractions from the presented problem. More precisely, the task was to subtract a random 1-digit number from a randomly selected number between 15–30 (e.g. 27-6 = 21, 21-6 = 15, etc).
- Spatial navigation (NAV): Imagination of navigating through a familiar house (flat) thereby focusing on orientation.
- Motor imagery of the right hand (HAND): Kinesthetic imagination of repetitively squeezing a hand-sized ball with the own right hand.
- Motor imagery of both feet (FEET): Kinesthetic imagination of repetitive self-paced movements of both feet without any actual movement.

Participants were seated about 0.7 m in front of a 17 inch computer monitor. Before each experiment began, participants received instructions on the task to be performed (both in writing as slideshow and verbally) and were asked to relax, avoid movements and reduce blinking during the experiment. To get acquainted with the experimental paradigm, subjects were asked to perform one exercise run consisting of two trials per class before the recording of imagery trials started. After the experiment participants were asked about the quality of their imagery, and the ease and enjoyment of mental tasks (5-point rating scale). Results on the questionnaires are presented elsewhere [48].

#### Data Analysis

Recorded EEG signals were visually scored by an expert and trials contaminated with muscle or eye movement activity within the imagery period were excluded from further analysis. Furthermore, noisy channels or channels severely contaminated with artifacts were excluded. Different channels were affected on either of the two days. To keep the information for single-trial classification identical on both days, affected channels were removed from both days. However, due to this procedure the number of EEG channels and the number of trials included in the analysis was different for each subject. Please see Table 1 for a detailed list of channels/trials that were removed/excluded.

The procedure applied in [40] for comparing within- and between-day variability in able bodied individuals was utilized. All possible mental task pair combinations were analyzed separately. The well established method of common spatial patterns (CSP) was used to design class specific spatial filters in the 8–30 Hz frequency band, and Fisher's linear discriminant analysis (LDA) classifier was used to classify the log-transformed normalized variance from 4 projections (m = 2) [54]. The CSP method projects multi-channel EEG data segments from two classes into a low-dimensional spatial subspace in such a way that the variances of the time series are optimal for discrimination ([55], for a tutorial see [56]). Designing CSP filters involves two steps. The first step is to whiten the EEG, i.e., to transform the distribution of the EEG into a spherical Gaussian distribution with covariance matrix Σ = *σ* ^2^ ⋅ *I*, where *I* denotes the identity matrix. The second step is to align the variances *σ* ^2^ projected along the principal axes with the coordinate axes (rotation). Only the most discriminative projections *Z*_*i*_, *i* = 1, …, 2*m*, obtained from the m-largest and m-smallest eigenvalues of the principal axes, are used for classification. For each projection *Z*_*i*_ the variance of the projection within the analyzed segment is computed and normalized by division by the sum of the variances of the 2*m* projections. Features used for classification are computed by taking the logarithm of the normalized values. Fisher’s LDA classifier is a linear hyper-plane classifier. LDA projects high-dimensional data onto a line and performs classification by thresholding in the projected one-dimensional space. The projection maximizes the distance between the means of the two classes while minimizing the variance within each class. See [57] for more details on LDA.

#### Within-day variability analysis

Each day was analyzed separately to rank the discriminability of the imagery pairs and to evaluate the within-day variability. To get an overview of timing and dynamics of the induced EEG patterns, trials were subdivided into thirteen 1-s data segments with 0.5 s overlap (*S*_*t*_ = [*t* − 1 *t*], *t* = 3.0, 3.5, …, 9.5*s*). For each *S*_*t*_ and imagery pair, CSPs and LDA were computed and evaluated using a 10-times 10-fold cross-validation statistic. To favor a balanced classification performance, we ranked the imagery pairs based on the geometric mean accuracy $GMAC=\sqrt{TPR\cdot TNR}$ and not on the arithmetic mean accuracy.

#### Between-day variability Analysis

BCI simulations were computed to assess the between-day variability. For every 1-s time segment *S*_*t*_ = [*t* − 1 *t*]*s*, *t* = 3.0, 3.5, …, 9.0*s*, within the imagery period of day one, CSP and LDA methods were trained and applied to day two. We typically use 1-s segments for classification because this segment length results in a reasonable tradeoff between classification accuracy and system reaction time during on-line feedback control [58]. To account for non-stationarities in EEG due to possible differences in the electrode montage and impedances between days and other noise sources, the first 4 trials of each class from day two were used to update the bias of the LDA [47]. CSP filter and LDA weights were not modified. Corresponding to on-line BCI signal processing, CSP filters were applied to 1-s EEG segments and classified by the re-biased LDA classifier. For comparison reasons, only EEG segments with a 0.5 s time-lag (1-s segment length) as used in the within-day variability analysis above were calculated.
